# Complete Genome Sequences of Human Respiratory Syncytial Virus Genotype A and B Isolates from South Korea

**DOI:** 10.1128/genomeA.00332-15

**Published:** 2015-04-23

**Authors:** Mi-ran Yun, A-Reum Kim, Han Saem Lee, Dae-Won Kim, Wan-Ji Lee, Kisoon Kim, Sung Soon Kim, You-Jin Kim

**Affiliations:** a Division of Biosafety Evaluation and Control, Korea National Institute of Health, Cheongju-si, Chungbuk, Republic of Korea; b Division of Respiratory Viruses, Center for Infectious Diseases, Korea National Institute of Health, Korea Center for Disease Control and Prevention, Cheongju-si, Chungbuk, Republic of Korea; c Division of Influenza Virus, Center for Infectious Diseases, Korea National Institute of Health, Korea Center for Disease Control and Prevention, Cheongju-si, Chungbuk, Republic of Korea

## Abstract

There is a paucity of complete genome sequence information for human respiratory syncytial virus (HRSV). To this end, we sequenced the complete genome sequences of HRSV genotype A (HRSV-A/IC688/12) and genotype B (HRSV-B/GW0047/14 and HRSV-B/IC0027/14). This information will increase the understanding of HRSV genetic diversity, evolution, pathogenicity, antigenicity, and transmissibility.

## GENOME ANNOUNCEMENT

Human respiratory syncytial virus (HRSV) is a main cause of lower respiratory tract infections, such as bronchiolitis and pneumonia, in neonates, children, and elderly people (1). It belongs to the *Pneumovirus* genus within the family *Paramyxoviridae* and has an enveloped, negative-sense, single-stranded RNA genome of approximately 15.2 kb. Eleven viral proteins are encoded in the following order: NS1, NS2, N, P, M, SH, G, F, M2-1, M2-2, and L. HRSVs are divided into two subgroups, A and B, based on sequencing and monoclonal antibody reactions against attachment (G) and fusion (F) glycoproteins (2). Currently, 14 HRSV-A genotypes (GA1 to GA7, SAA1, CB-A, NA1 to NA4, and ON1) and 22 HRSV-B genotypes (GB1 to GB4, SAB1 to SAB4, URU1 to URU2, and BA1 to BA12) have been designated (3). Since 1999, a duplication of 60 nucleotides (nt) in the C terminus of the G gene in HRSV-B has been identified; this BA genotype has completely replaced non-BA genotypes (4). In the 2010–2011 winter season, HRSV-A strains with a 72-nt duplication (ON1 strain) at the C-terminal end were discovered in Canada (5). Subsequently, several countries (South Korea, China, Italy, Japan, Germany, Malaysia, and South Africa) reported ON1 dissemination, and its rapid spreading was documented in South Korea (6, 7).

In this study, we determined one complete genome sequence of HRSV-A (HRSV-A/IC688/12), isolated in July 2012 from an infant less than 1 year old, and two complete genomes of HRSV-B (HRSV-B/GW0047/14 and HRSV-B/IC0027/14), isolated in January 2014 from a 45-year-old and a 1-year-old acute respiratory illness patient, respectively. The three HRSVs were isolated from acute respiratory illness patients using the HEp-2 cell line, and plaque isolation was performed twice. Then, cultured viral isolates were deposited in the National Culture collection for Pathogens (http://nccp.cdc.go.kr) in South Korea. First-strand cDNA was synthesized from the viral culture with random primers, and full-length genomes were obtained from 11 fragment PCR products using an ABI 3730XL DNA analyzer with 25 fragment-specific and inner primers (6).

The genome of strain HRSV-A/IC688/12 was 15,172 bp in length and belonged to the NA1 genotype based on its G gene sequence, which lacked the 72-nt duplication. HRSV-B/GW0047/14 and HRSV-B/IC0027/14 genome sequences were BA4 genotype and 15,235 bp in length with a 60-nt duplication in the C terminus of the G protein gene. Sequence homology between these full-length genome sequences and the reference sequences, HRSV A2 (GenBank accession number M74568) and HRSV-A/IC688/12, was 94.88% at the nucleotide level. Comparison of full-length nucleotide sequences between strain RSV-A/NIV1114073/11 (8) and strain HRSV-A/IC688/12 revealed a very high similarity of 99.4%. Both HRSV-B/GW0047/14 and HRSV-B/IC0027/14 full-length nucleotide sequences have 99.3% identity with the full-genomic sequence of the HRSV-B strain, B/WI/629-12/06-07 (9). Overall G+C contents were 33.27, 33.60, and 33.64% for HRSV-A/IC688/12, HRSV-B/GW0047/14, and HRSV-B/IC0027/14, respectively.

### Nucleotide sequence accession numbers. 

The complete genome sequences of the HRSV-A/IC688/12, HRSV-B/GW0047/14 and HRSV-B/IC0027/14 isolates were submitted to GenBank under the accession numbers KP663728 to KP663730.
